# The Influence of Menstrual Cycle Phase and Urinary Incontinence on Potential ACL Injury Risk Factors with a Focus on Hip Strength and Postural Control in Elite Female Team Sport Athletes: A Pilot Study

**DOI:** 10.3390/sports14030096

**Published:** 2026-03-03

**Authors:** Elisabeth Maria Kirschbaum, Roxane Windisch, Katrin Heyde, Richard Hunger, Kirsten Legerlotz

**Affiliations:** 1 Movement Biomechanics, Institute of Sport Science, Humboldt-Universität zu Berlin, 10115 Berlin, Germany; legerlotz@uni-wuppertal.de; 2 Research Group Field Hockey, Institute for Applied Training Science, 04109 Leipzig, Germany; katrin.heyde@sport-iat.de; 3 Department of Sport and Sport Science, Sport Psychology, Albert Ludwig University Freiburg, 79102 Freiburg, Germany; 4 Statisticum Simplex—Statistical Consulting, 04105 Leipzig, Germany; statistik@fn.de; 5 Department of Movement and Training Science, Institute of Sport Science, University of Wuppertal, 42119 Wuppertal, Germany

**Keywords:** balance, symptoms, women, hormones

## Abstract

To improve understanding of anterior cruciate ligament (ACL) injuries, this study investigated the effect of menstrual cycle (MC) phase on ACL injury risk factors in elite female team sport athletes with and without urinary incontinence (UI). Additionally, associations between endogenous sex hormones, MC-related symptoms, and these risk factors were investigated. Ten elite female athletes (24.2 ± 3.6 years, BMI 23.2 ± 1.3 kg/m^2^, 10.9 ± 1.8 training hours/week) completed three testing sessions across three MC phases, determined using the three-step method. Assessments included static and dynamic postural control and hip strength. Mixed-model ANOVA and canonical correlation analyses evaluated the effects of MC phase, UI, hormones, and performance. A significant interaction between MC phase and UI was observed for single-leg sway area with eyes closed (*p* = 0.036), and UI was associated with a higher hip adduction:abduction ratio (*p* = 0.037). No further significant interaction between UI and MC phase was observed. Moreover, hormones explained 16.5% of the variance in risk factors, while subjective symptoms explained 24.5%. Lower progesterone was associated with higher symptoms, lower estradiol and progesterone with reduced strength and poorer postural control, and higher testosterone with greater strength. Although limited by its pilot design, menstrual symptoms, more than MC phases, may influence performance and injury risk, supporting the potential value of systematic symptoms monitoring.

## 1. Introduction

Anterior cruciate ligament (ACL) injuries are a major concern in sports, with women having an up to eightfold higher risk compared to men [1]. The ACL injury risk is affected by multiple variables involving biomechanical and anatomical factors, many of which are sex-specific [2]. In addition to anatomical and biomechanical differences, hormonal fluctuations during the menstrual cycle (MC) are frequently discussed as potential markers influencing injury risk [3,4]. In particular, the follicular phase and the days around ovulation are considered phases of increased injury susceptibility [5,6], potentially due to MC-related hormonal influences on joint stability and neuromuscular control [2,7]. However, evidence for increased ACL injury risk during the follicular phase and around ovulation, as well as for associated biomechanical and neuromuscular risk factors, remains limited due to poor-quality MC assessment and study design [5,8]. While associations between the MC phase and ACL injury have been observed, the causal relationship remains unclear [7]. Among (neuro-)muscular risk factors for ACL injuries, decreased hip abduction strength is well-established, whereas evidence for reduced postural control is less consistent [4,9].

Moreover, the pelvic floor muscles may affect ACL injury risk by modulating both postural control and hip muscle function, as the female pelvic floor muscles are essential for core stability and are therefore coactivated during functional movements [10]. Hip abductors and adductors, as well as pelvic floor muscles, exhibit synergistic contraction; thus, maximal activation of one muscle group can affect the others [11]. Weak or absent co-contraction, for example, during hip movements, may contribute to urinary incontinence (UI) [12], a condition prevalent among elite female athletes, particularly in high-impact sports, such as ball games, with reported rates of 26% [13]. UI may further compromise concentration, performance [14], and postural control [15]. Furthermore, there may be an interaction between the MC phase and UI, as, e.g., ovulation is associated with a moderate UI increase [16]. While this aligns with the time point of increased ACL injury susceptibility [5], other risk factors show different associations with specific phases of the MC. Taken together, these observations suggest a hypothetical pathway in which pelvic floor function, the MC phase, and UI could influence ACL injury susceptibility.

Initial studies suggest that hip abduction strength may be reduced during the early follicular phase (EFP) [17], whereas postural control, particularly in women with premenstrual syndrome (PMS), may be reduced during the late luteal phase [18]. Since ACL injuries arise from multiple interacting factors rather than a single cause [4,7], it is important to study several contributing variables simultaneously to uncover their interactions, improve understanding of injury, and guide more effective prevention strategies, particularly in elite sports where data remain scarce.

Accordingly, the aim of this study was to examine the effect of MC phase on ACL injury risk factors, specifically postural control and hip strength, in elite female team sport athletes with and without UI, using gold-standard methods for MC phase determination. While this study does not measure ACL injuries directly, it focuses on established neuromuscular and biomechanical risk factors associated with increased ACL injury susceptibility. The secondary aim was to investigate the association between endogenous sex hormones, MC-related symptoms, and risk factors.

## 2. Materials and Methods

### 2.1. Study Design and Setting

**Figure 1:**
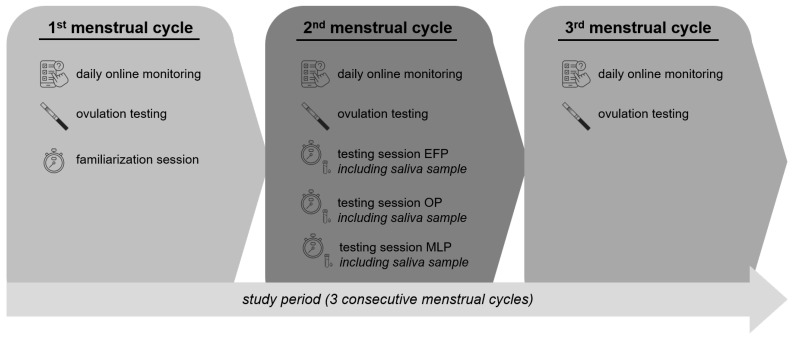
Schematic overview of the study design. EFP, early follicular phase; OP, ovulation phase; MLP, mid-luteal phase.

A prospective longitudinal observational design was chosen to investigate the relationship between hip strength and static, as well as dynamic balance, across a single MC in elite female athletes (Figure 1: Schematic overview of the study design). Data from three consecutive MCs were collected. In the first MC, participants conducted MC monitoring, followed by a familiarization session at the end of the first MC to acquaint participants with the procedures and requirements of the testing protocol. During the second MC, besides MC monitoring, testing was conducted at three predefined time points within the MC: EFP (MC days 2–5), ovulation phase (OP, within 48 h of a positive ovulation test), and mid-luteal phase (MLP, 7–9 days after a positive ovulation test) [19]. In the third MC, only MC monitoring was performed.

Prior to participation, all participants received detailed information about the study procedures and objectives and provided written informed consent. The study was conducted in accordance with the Declaration of Helsinki and was reviewed and approved by the Ethics Review Board of the Institute for Applied Training Science (ER_2024.29.12_2).

### 2.2. Participants

**Figure 2:**
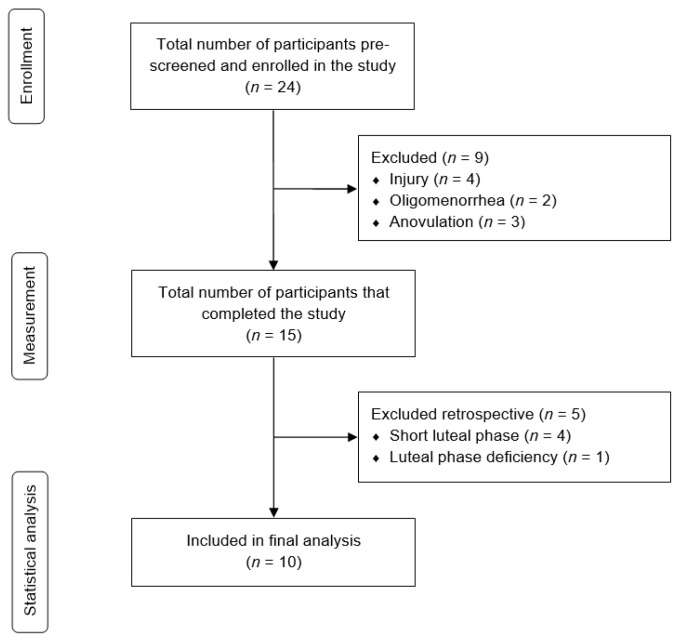
Flow chart of participant inclusion. Exclusion due to MC dysfunction corresponded to the step of the “three-step method” in which it was identified. In step 1, oligomenorrhea was detected via diary monitoring (*n* = 2), in step 2, anovulation and short luteal phase were detected via ovulation testing (*n* = 7), and in step 3, luteal phase deficiency was identified via hormonal verification (*n* = 1).

Female athletes were eligible for this study if they met the following criteria: (1) Tier 4 athletes [20], corresponding to a current competitive level in Germany of at least the 2nd national division (football and handball), 1st national division (basketball and field hockey), or national team level (lacrosse and rugby), (2) age ≥ 18 years, (3) no use of hormonal contraceptives for at least three months prior to participation in the study, (4) an average MC length of 21–35 days, (5) nulliparity, (6) no serious lower-extremity injuries within the past six months, and (7) no neurological or musculoskeletal disorders that could affect balance. Recruitment was conducted in person with eight potentially eligible teams. Data collection was conducted in Hamburg from February to July 2024 and in Leipzig from January to June 2025. A total of 24 elite athletes were initially enrolled. During the study period, however, four participants were excluded due to injuries, and ten were excluded due to MC dysfunctions. Consequently, the final sample comprised ten athletes from field hockey (*n* = 7), football (*n* = 2), and lacrosse (*n* = 1) (Figure 2).

### 2.3. Stress Urinary Incontinence

To assess UI, the German and English versions of the International Consultation on Incontinence Questionnaire–Urinary Incontinence Short Form (ICIQ-UI SF) were used [21]. Participants were classified as having UI if they scored ≥ 1 point on the questionnaire [22] in order to capture the presence of any symptoms, including very mild cases, that still may have an impact in the context of elite sports. UI was assessed at a single time point in this study.

### 2.4. Menstrual Cycle Monitoring

MC phases were determined using the “three-step method” [23]. In the first step, participants recorded the first day of their menstruation in a diary and completed a daily online questionnaire (Version 3.4.22, SoSci Survey GmbH, Munich, Germany), assessing the presence and heaviness of menstrual bleeding, medication use, training load, sleep, well-being, and symptoms using a validated German DSM-IV-TR–based PMS questionnaire [24], with translation aids provided in participants’ native languages for non-German speakers (File S1: Daily menstrual cycle monitoring questionnaire in English and German). In the second step, participants performed daily morning digital ovulation tests (Clearblue, SPD Swiss Precision Diagnostics GmbH, Geneva, Switzerland), beginning on the MC day recommended by the manufacturer and continuing until either a positive result was obtained or menstruation began. The day of ovulation was defined as the first day with a positive ovulation test result. MC length was calculated as the number of days from the first day of menstruation to the day before the next menstruation. The luteal phase length was determined as the number of days from the day after ovulation to the day before the next menstruation [25]. In the third step, saliva samples were collected at the beginning and end of each testing session to verify the MC phases (EFP, OP, and MLP). Samples were immediately frozen at −20 °C and later analyzed for estradiol, progesterone, and testosterone concentrations. To ensure accurate measurements, participants were instructed to refrain from eating, drinking (except water), and smoking for at least 60 min prior to sample collection.

MCs were subsequently classified retrospectively as either eumenorrheic or dysfunctional. MC dysfunction was defined as (1) oligomenorrhea (MC length > 35 and <90 days), (2) anovulation (no positive ovulation test), (3) a short luteal phase (<10 days), or (4) luteal phase deficiency (saliva progesterone concentration < 50 pg/mL and <1.5× EFP [26]) [19,25]. MCs in which any of these abnormalities were detected were excluded from the analysis (Figure 2). All remaining MCs were classified as eumenorrheic and included in the analysis.

### 2.5. Test Protocols

#### 2.5.1. Familiarization Session

This session was identical in structure and execution to the subsequent testing sessions. In addition, anthropometric data, including body height and leg length, were collected during this session. The values obtained during familiarization served as reference measures for device-specific warm-up procedures during hip strength testing in the test sessions.

#### 2.5.2. Test Day Procedures

**Figure 3:**
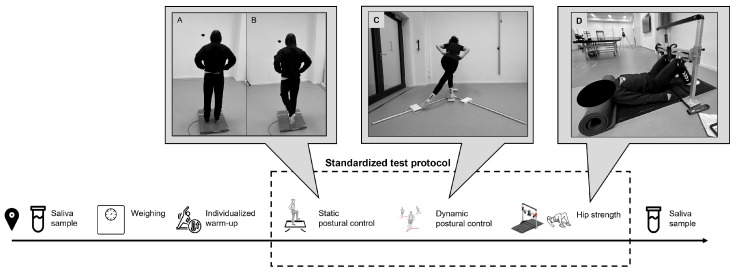
Illustration of test day procedures. (**A**) Starting position during measurement of static postural control in bipedal stance and (**B**) in single-leg stance. (**C**) Dynamic postural control and (**D**) procedure for hip strength measurement.

Upon arrival, participants were first asked to provide an initial saliva sample. They were then weighed and asked to perform an individual warm-up. Since the participants were elite athletes, we decided not to prescribe a standardized warm-up, as they came from different sports and clubs and already had their own routines. Allowing athletes to perform warm-ups they perceive as most beneficial accounts for inter- and intra-individual differences and ensures they are optimally prepared and focused for subsequent performance testing [27]. After warming up, they were asked to empty their bladder, and testing proceeded with the assessment of static postural control, followed by dynamic postural control and hip strength (Figure 3). Upon completion of the hip strength tests, the second saliva sample was collected. Each testing session was conducted at approximately the same time of day (±1 h) for each participant to minimize diurnal variations in the results. The same examiner conducted all assessments throughout the study.

##### Static Postural Control

Static postural control was assessed using a force plate (Kistler Instrumente AG, Winterthur, Switzerland). Data were recorded with BioWare software (Version 5.3.0, Kistler Instrumente AG, Winterthur, Switzerland) at a frequency of 200 Hz. The plate was calibrated before each session. Participants performed barefoot 30 s trials of bipedal and single-leg stances with eyes open (EO) and closed (EC), following Fridén et al. [18], with a one-minute rest between trials in a quiet room. Trial order was: bipedal EO, bipedal EC, right single-leg EO, left single-leg EO, right single-leg EC, left single-leg EC. This fixed order standardized progression from easier to more challenging stances and ensured participant safety.

Feet were parallel in a bipedal stance, and in the single-leg stance, the non-supporting knee was flexed (Figure 2). Hands were on the hips, and a fixation point ~2 m away was used for EO; EC trials focused on the same point prior to eye closure. Trials were repeated if participants stepped off the plate, touched down with the non-supporting leg, or opened their eyes during EC. If balance was lost in the last 10 s, analysis included data up to 2 s before contact.

Center of pressure data were used to quantify static postural control via total sway path and 95% sway area [28].

##### Dynamic Postural Control

Dynamic postural control was assessed using the Y-Balance Test (Functional Movement Systems Inc., Chatham, VA, USA). Participants performed the test barefoot, standing on the tested foot with their hands on the hips, and reached with the free leg in the anterior (A), posteromedial (PM), and posterolateral (PL) directions. Three valid trials per direction and leg were recorded, with one practice trial allowed. The standardized testing order was: A left, A right, PM left, PM right, PL left, PL right, alternating stance legs between directions to minimize fatigue.

Trials were repeated if balance was lost, the stance foot or heel lifted, contact with the reach indicator was lost or used for stabilization, or the starting position was not controlled [29]. Maximal reach distances and number of failed attempts were recorded. Distances were normalized to individual leg length, measured supine from the anterior superior iliac spine to the distal medial malleolus.

For analysis, the A reach difference and Composite Reach Score (CRS) were calculated [30]. CRS was determined separately for each leg, and the mean of both legs was used as the overall CRS.

##### Hip Strength

Isometric hip muscle strength was assessed using the GroinBar (VALD Performance, Albion, Australia). Participants lay supine with 45° hip flexion and approximately 90° knee flexion. Arms were extended alongside the body on the floor, and feet were positioned hip-width apart (Figure 2). For sensor placement, sensors were positioned medially at the femoral condyles for adduction and laterally for abduction. The height of the GroinBar was adjusted to align the sensor centers with the knee joints and maintained throughout testing. All settings were documented to ensure identical positioning in subsequent sessions. Joint angles were checked before and during each trial using a digital goniometer (Digital Goniometer Baseline^®^ Absolute Axis 360°, Model 1013990, Fabrication Enterprises Inc., White Plains, NY, USA) to avoid deviations from the starting position [31].

For warm-up, participants performed two submaximal contractions at 80% of maximal voluntary contraction (F_max_) per muscle group. This was followed by three sets of alternating maximal isometric contractions of the hip adductors and abductors, each held for 5 s with a visible force plateau. A minimum rest of 10 s was provided between contractions [32]. Constant verbal encouragement was given throughout testing.

For each muscle group and trial, F_max_ was defined as the best single value recorded both in absolute terms [N] and relative to body mass [N/kg]. F_max_ was automatically determined using the accompanying software (Version 2.0.1, ForceFrame App, VALD Performance, Albion, Australia).

### 2.6. Statistical Analysis

All statistical analyses were performed using MS Excel (Microsoft Corp., Redmond, WA, USA), IBM SPSS Statistics for Windows, Version 29 (IBM Corp., Armonk, NY, USA), and RStudio, Version 3.6.0 (Posit Software, Boston, MA, USA). Data are presented as mean ± standard deviation or median (IQR). To examine the effect of UI, a mixed-model repeated-measures ANOVA was conducted with one within-subject factor (MC phase: EFP, OP, MLP) and one between-subject factor (UI vs. non-UI). This analysis assessed the main effects of MC phase and UI, as well as their interaction. Differences between MC phases in hormonal profiles, training load, sleep, and well-being were analyzed using repeated-measures ANOVA when the assumptions of normality (Shapiro–Wilk test) and sphericity (Mauchly’s test) were met. When the normality assumption was violated, the nonparametric Friedman test was used. In cases of sphericity violation, the Greenhouse-Geisser correction was applied. Post hoc tests with Bonferroni correction were performed to identify significant differences in main effects. Effect sizes were expressed using partial eta-squared (η_p_^2^) for ANOVA and Kendall’s ω for the Friedman test. In accordance with Cohen [33], η_p_^2^ values of ≥0.01, ≥0.06, and ≥0.14 were interpreted as small, medium, and large effects, respectively, while corresponding thresholds for Kendall’s ω were ≥0.10, ≥0.30, and ≥0.50 [33]. Due to the small sample size, the following ANOVA results should be interpreted as exploratory. Statistical significance is reported to aid interpretation of observed association patterns but should not be considered confirmatory.

For the secondary aim, a canonical correlation analysis (CCA) [34] was conducted to examine associations between endogenous sex hormone levels, MC-related symptoms, and risk factors. The variables entered into the CCA were selected a priori based on their theoretical relevance to MC-related symptoms, perceived well-being and neuromuscular performance. To limit model complexity relative to sample size, only summary metrics (e.g., total sway path, sway area, relative strength measures) were included rather than multiple highly correlated derivatives. Three sets of variables were defined: the first set included endogenous sex hormones (estradiol, progesterone, testosterone and the progesterone:estradiol ratio); the second set comprised subjectively perceived MC-related perceptual and symptom variables (overall symptom score, overall well-being score, and session rating of perceived exertion (sRPE)); and the third set included risk factors (variables from the static postural control, the dynamic postural control and the hip strength assessment). Three pairwise CCAs between each variable set were performed. For each canonical function, we computed canonical coefficients, structure coefficients (r), canonical correlations (r_cc_), and redundancy indices (R^2^). As a directed association is hypothesized, only redundancy measures in the respective causal relationship are reported, using the following interpretation thresholds: R^2^ < 0.10, weak redundancy; 0.10 ≤ R^2^ ≤ 0.25, intermediate redundancy; and R^2^ > 0.25, strong redundancy. Significance of the canonical correlations was assessed using complex survey CC, an adjusted test that incorporates the repeated measurement design. Only statistically significant canonical functions were reported and interpreted. Interpretation of variable contributions was based primarily on structure coefficients, using |r| ≥ 0.30 as the threshold for meaningful loadings. A CCA was chosen to explore multivariate association patterns between perceptual/symptom-related variables and neuromuscular and postural control measures, as these domains are theoretically interdependent and unlikely to be adequately represented by isolated univariate or bivariate analyses, which may have further inflated the number of comparisons and the likelihood of Type-I errors. Despite the small sample size, CCA was deemed suitable for hypothesis-generating purposes, allowing the identification of shared variance structures across variable sets that may inform future confirmatory studies. Canonical coefficients and redundancy indices should be interpreted with caution, as they are likely to be sample-specific and may not replicate even in moderately larger cohorts. In addition, canonical correlations are known to be upwardly biased and may reflect overfitting, and therefore, interpretation focused primarily on the redundancy indices, which provide a more conservative estimate of shared variance between the variable sets and reduce the risk of overstating associations based solely on the magnitude of r_cc_.

Given the small final sample size, all statistical analyses were conducted with an awareness of limited statistical power and an increased risk of Type I error. Consequently, the mixed-model ANOVA and CCA were intended to explore multivariate association patterns rather than to provide confirmatory inference. Effect sizes and multivariate relationships were therefore prioritized over null-hypothesis significance testing.

For all analyses, a *p*-value ≤ 0.05 was considered statistically significant.

## 3. Results

### 3.1. Participant Characteristics, MC Parameters and Hormonal Profiles

The athletes were on average 24.2 ± 3.6 years old and had a BMI of 23.2 ± 1.3 kg/m^2^. They had been practicing their sport for an average of 16.3 ± 5.0 years and reported a weekly training volume of 10.9 ± 1.8 h. The age at menarche was 13.0 ± 1.5 years. Four athletes were classified as having UI. The average score in the ICIQ-UI SF in this group was 4.3 ± 1.3 (Min: 3; Max: 6) (Table 1).

The average MC length was 27.9 ± 2.0 days (Min: 25 days; Max: 32 days), and the luteal phase lasted an average of 13.3 ± 1.6 days (Min: 11 days; Max: 16 days). Measurements in the EFP were taken between day 2 and 5 of the MC, most frequently on day 3 (*n* = 4). Measurements in the OP were most frequently taken two days after a positive ovulation test (*n* = 6), which was between day 13 and 19 of the MC. Measurements in the MLP were most frequently taken on day 7 after ovulation (*n* = 7), corresponding to the period between day 20 and 26 of the MC.

The sex hormone concentrations measured for each phase were reflective of eumenorrheic MC. There was a significant main effect of MC phase on progesterone (*Χ*^2^(2) = 18.200, *p* < 0.001, *n* = 10) and on the progesterone:estradiol ratio (F(2, 18) = 32.278, *p* < 0.001, η_p_^2^ = 0.782). Post hoc tests revealed a significantly higher progesterone concentration in the MLP compared to the EFP (*p* < 0.001) and the OP (*p* = 0.014), while EFP and OP did not differ significantly (*p* = 0.074). The progesterone:estradio ratio was significantly higher in the MLP compared to the EFP (*p* < 0.001) and the OP (*p* = 0.001), and in the OP compared to the EFP (*p* = 0.026) (Table 2).

### 3.2. Effects of MC Phase and UI on ACL Risk Factors

The median values of static and dynamic postural control and hip strength are presented in Table 3, while individual patterns of single participants are presented in Figure S1. The mean within-participant coefficient of variation across MC phases for all variables was 15.4 ± 4.1% (Min: 7.9; Max: 20.4). In total, one statistically significant interaction between MC phase and UI was identified, as well as one significant main effect of the between-subjects factor UI (Table 3).

A significant interaction between the repeated-measures factor MC phase and the between-subjects factor UI was observed for static postural control in the total sway path during the single-legged condition with EC (F(2, 16) = 4.142, *p* = 0.036, η_p_^2^ = 0.341). Post hoc analyses revealed no significant differences between the UI and non-UI group during the EFP, OP, or MLP. Within-group comparisons showed no significant phase differences in the UI group, whereas the non-UI group demonstrated a significantly lower total sway path in the OP compared with the MLP (*p* = 0.008), with no significant differences between the MLP and EFP (*p* = 0.896) or between the OP and EFP (*p* = 0.055). Additionally, the between-subjects factor UI was significantly associated with the adduction:abduction ratio of hip strength, with higher values observed in the UI group (*p* = 0.037). For all other variables, no significant interaction effects of UI and MC phase, between-subjects factor UI, or within-subjects factor MC phase were observed (*p* > 0.050).

### 3.3. Interaction Between Symptoms, Hormone Levels and Risk Factors

**Figure 4:**
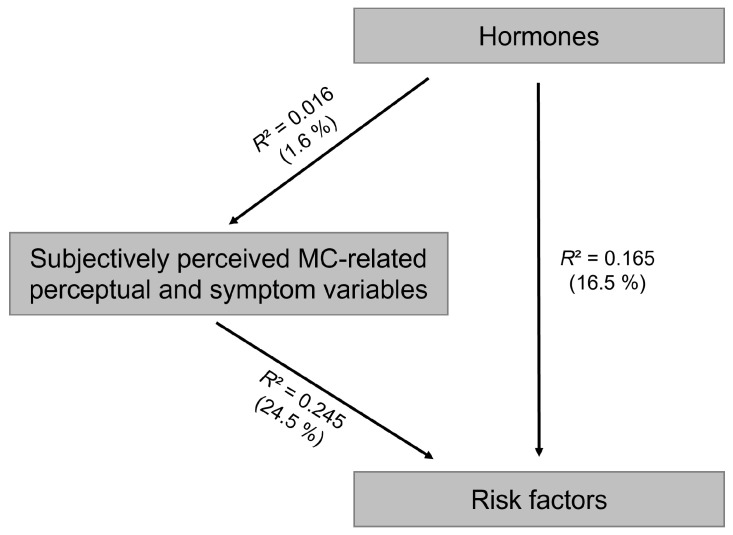
Schematic representation of the results of the canonical correlation analysis. Mean proportion of variance (R^2^) in each variable set explained by canonical correlation analysis (CCA) between the three sets: hormones, subjectively perceived MC-related perceptual and symptom variables, and risk factors.

Regarding symptoms, training load, sleep, and well-being, no interaction with MC phase was observed (*p* > 0.050) (Table 2). Three CCAs were conducted between the three variable sets. The first CCA examined the association between the hormone set and the subjectively perceived MC-related perceptual and symptom variables set. The hormone set explained on average 1.6% of the variance in the subjective MC-related variables. The second CCA examined the association between the subjective MC-related variables set and the risk factor set, with the subjective MC-related set explaining 24.5% of the variance in the risk factor variables. The third CCA assessed the association between the hormone set and the risk factor set. Here, the hormone set explained 16.5% of the variance in the risk factor variables (Figure 4).

In the first CCA, one canonical function was statistically significant (F(29, 8) = 3.750, *p* = 0.006, r_cc_ = 0.681, R^2^ = 0.045). The results indicate an inverse relationship between progesterone (r = −0.445) and the overall symptom score (r = 0.403), suggesting that lower progesterone levels might be associated with higher symptom severity.

In the second CCA, three significant canonical functions were observed between the subjectively perceived MC-related perceptual and symptom variables set and the risk factor set. The first function (F(26, 7) = 7.606, *p* < 0.001, r_cc_ = 0.884, R^2^ = 0.271) was primarily characterized by a pattern in which higher well-being (r = 0.793) might be associated with greater relative adduction strength (r = 0.453), reduced total sway path (r = −0.420), and decreased sway area during the single-leg stance with EC (r = −0.498). The second function (F(26, 7) = 7.613, *p* < 0.001, r_cc_ = 0.864, R^2^ = 0.155) was most strongly associated with overall well-being score (r = 0.490) and sRPE (r = −0.465), as well as the A difference (r = 0.487). In the third function (F(26, 7) = 6.726, *p* < 0.001, r_cc_ = 0.830, R^2^ = 0.271), the overall symptom score (r = −0.812) and total sway path in bipedal stance with EO (r = 0.439) were the variables with the largest canonical coefficients. Across all three functions, canonical coefficients and structure coefficients did not consistently align with theoretical expectations.

In the third CCA, four significant canonical functions were detected between the hormone variables set and the risk factor variables set. The first function (F(26, 7) = 30.900, *p* < 0.001, r_cc_ = 0.969, R^2^ = 0.208) was primarily characterized by estradiol (r = −0.722), along with absolute adduction strength (r = −0.400), adduction:abduction ratio (r = −0.451), and total sway path in the single-leg stance with EC (r = 0.415), indicating that lower estradiol levels might be associated with poorer performance on these risk factors. The second function (F(26, 7) = 6.885, *p* < 0.001, r_cc_ = 0.821, R^2^ = 0.119) was dominated by testosterone (r = 0.788) and absolute adduction strength (r = 0.308), suggesting that higher testosterone levels might be associated with greater strength. In the third function (F(26, 7) = 6.037, *p* = 0.001, r_cc_ = 0.751, R^2^ = 0.017), the largest canonical coefficients were for progesterone (r = −0.953), relative adduction strength (r = −0.390), and total sway path in bipedal stance with EC (r = 0.336), indicating that lower progesterone levels might be associated with lower strength and poorer postural control. The fourth function (F(26, 7) = 3.072, *p* = 0.018, r_cc_ = 0.538) was characterized by the progesterone:estradiol ratio (r = 0.461), relative adduction strength (r = −0.332), and sway area in bipedal stance with EC (r = 0.318), suggesting that a higher progesterone:estradiol ratio might be associated with lower strength and poorer static postural control.

## 4. Discussion

As a pilot study with a small sample of elite female athletes, the present findings are exploratory and intended to generate hypotheses rather than provide definitive conclusions about ACL injury risk factors across MC phases. This study highlights that while MC phase alone has limited direct effects on most ACL injury risk factors, symptom severity and hormone levels, particularly progesterone and estradiol, show potential associations with postural control and hip strength, suggesting that individual physiological and symptomatic profiles may be more relevant for athlete assessment and training adaptation than MC phase alone.

### 4.1. UI and ACL Injury Risk

Athletes with UI exhibited a significantly higher adduction:abduction ratio, indicating relative hip abduction weakness, a known risk factor for ACL injury [9]. This risk may be further influenced by the pelvic floor muscles, which coactivate with the hip and core musculature to support postural control [10,11]. Insufficient pelvic floor muscle contraction can contribute to UI [12], which may therefore indirectly affect balance and hip strength, although this was not apparent in our cohort due to the mild severity of UI observed. Specifically, three athletes presented with mild UI (ICIQ-UI SF scores 3–4) and one athlete with moderate UI (score 6) [22], and therefore, the findings may not be generalizable to athletes with more severe UI. The absence of athletes with severe UI may also be explained by the inclusion criteria, which restricted MC length to 21–35 days, thereby excluding athletes with oligomenorrhea or secondary amenorrhea. UI has been proposed as a potential component of the relative energy deficiency in sport (REDs) complex [35], although no significant association between UI and menstrual dysfunction has been reported [36]. However, in this study, 10 of 24 initially screened athletes were excluded due to subtle menstrual dysfunction, despite initially appearing eumenorrheic. One athlete was excluded due to luteal phase deficiency. The progesterone threshold used was adopted from a previous study [26]. However, salivary cut-offs for menstrual dysfunction remain debated and require further validation.

Future investigations should include a broader range of athletes, encompassing those with and without UI, as well as those with menstrual dysfunction or using hormonal contraception. Given that approximately one-third of team sport athletes use hormonal contraceptives [37], including this population would provide a more comprehensive understanding of the complex interactions between UI, hormonal profiles, and ACL injury risk in female athletes. Furthermore, UI in this study was assessed indirectly using the ICIQ-UI SF questionnaire. Although widely used and validated [21], this instrument does not provide a direct, objective evaluation of pelvic floor muscle function. More detailed and objective evaluations of pelvic floor muscle function, such as palpation or sonographic assessment, could yield a more accurate characterization of pelvic floor muscles [38,39]. It is plausible that more pronounced differences would emerge in athletes with more severe UI or when pelvic floor function is assessed directly, including across multiple MC phases rather than a single time point. Nevertheless, our findings suggest that pelvic floor muscle function could be an underrecognized contributor to injury risk in female athletes, warranting further investigation.

### 4.2. Neuromuscular Risk Factors for ACL Injury in Elite Female Athletes

In the present pilot study, only the risk factor of static postural control under the most challenging condition, single-leg stance with EC, showed a significant interaction between MC phase and the UI group. The total sway path differed significantly between MC phases in the group without UI, with the best values observed in the OP, which is generally considered the MC phase with the highest ACL injury risk [5]. However, it remains unclear to what extent static postural control is a meaningful predictor of ACL injury risk in elite team sports, as most ACL injuries occur during high-intensity, dynamic movements, such as rapid changes in direction or sudden deceleration, rather than during static tasks [40]. While postural control may represent an underlying factor contributing to injury susceptibility [4], static balance tests may lack sensitivity in elite athletes. Similarly, hip strength was assessed only isometrically, which may not fully capture dynamic, high-velocity movements that typically precede ACL injuries. Perturbation-based balance tasks could provide a more valid assessment of neuromuscular control by requiring active recovery of stability. However, such approaches have practical limitations: oscillatory platforms are typically laboratory-based, and perturbations are generally difficult to standardize across participants and sessions. As a feasible alternative, future research could focus on whole-body kinematics during dynamic movements, such as side-cutting or change-of-direction tasks, which may provide informative, field-applicable insights into neuromuscular control in elite athletes [41]. Overall, this suggests that both static balance tests and isometric strength assessments may not fully capture the dynamic neuromuscular demands relevant to ACL injury risk in elite female athletes, highlighting the need for protocols that reflect the neuromuscular demands of elite athletes.

### 4.3. MC Phases and Neuromuscular Performance

Neuromuscular changes during specific MC phases may not be directly causally related to increased injury risk. Although previous studies have reported a higher incidence of ACL ruptures during the follicular phase and around ovulation [5], the present pilot study showed that performance in relevant risk factors, including hip abduction and adduction strength as well as static and dynamic postural control, remained consistent across MC phases, with minimal improvements observed in some measures during OP or EFP. These phase-based observations were further supported by hormonal analyses.

Specifically, lower estradiol and progesterone concentrations, characteristic of the EFP and late luteal phase, as well as a higher progesterone:estradiol ratio, typical of the MLP [19], were associated with poorer performance on risk-related outcomes. Conversely, higher testosterone levels, which typically peak during the OP, were associated with better strength outcomes. Alternative explanations should therefore be considered. In particular, motivational and behavioral factors may play a central role. Female athletes are known to exhibit increased motivation and risk-taking behavior during the OP, which is also linked to elevated testosterone [42,43]. This could lead to altered behavior in game situations, for example, increased aggressiveness in tackles or an overestimation of performance, and thereby influence injury risk independently of the measured physical parameters.

### 4.4. Impact of Menstrual Symptoms on Performance and Injury Risk

Symptoms, rather than MC phase or hormone levels alone, appear to be most associated with performance and injury risk factors in elite female athletes. In the present pilot study, symptom burden accounted for the greatest variability in MC-related outcomes, supporting previous findings that symptoms exert a more meaningful influence than endogenous hormone fluctuations [44]. Notably, substantial intra- and inter-individual variability was observed, indicating that some athletes experienced minimal MC-dependent changes, whereas others showed pronounced fluctuations. From an applied perspective, this variability is encouraging, as symptoms can be directly targeted through athlete-centered monitoring and management [45]. It also highlights the limitations of generalized statements about MC-dependent performance and underscores the need for individualized assessment and prevention strategies [46].

Evidence from prior research further underscores the importance of managing symptoms, as unresolved or poorly managed symptoms can impair recovery and sleep, which in turn may influence ACL injury risk factors [47]. While training workload or fatigue alone have not been directly linked to ACL risk factors [48], poor sleep has been associated with detrimental effects on these outcomes [49]. Importantly, the MC itself should not be considered a primary marker of ACL risk. In women’s sports, long-term athletic development and regular assessment of general risk factors and neuromuscular imbalances are essential [50,51]. Systematic screening not only supports injury prevention but also provides benchmarks for return-to-play, enabling safe and effective reintegration into sport. Ongoing monitoring of training adaptation, together with tracking both external and internal loads, facilitates individualized, evidence-based injury-prevention strategies [52]. Consequently, incorporating MC and symptom monitoring into routine load management may offer a practical approach not only to optimize training and reduce injury risk but also to detect menstrual dysfunction and to support overall athlete health.

### 4.5. Strengths and Limitations

A strength of this study is its use of gold-standard methods for MC phase determination and objective assessments of ACL injury risk factors, enabling precise MC phase-specific comparisons. In addition, the inclusion of elite-level athletes enhances the applicability and relevance of the findings to high-performance sport. Although MC phase verification was robust, inferential conclusions remain limited by the small sample size and the complexity of the multivariate analyses. The strict three-step verification process for determining MC strengthens internal validity but may reduce ecological validity and limit representativeness within elite sport populations, as a substantial proportion of initially screened athletes were excluded due to subtle menstrual dysfunction (10 of 24).

A limitation of this pilot study is the small sample size (*n* = 10), which may introduce selection bias, limit the statistical power of the results and reduce their generalizability to the broader target population. Given the small sample size, the present findings, especially the results of the multivariate analyses, should be considered hypothesis-generating. The identified multivariate association patterns warrant replication in larger, adequately powered cohorts before firm conclusions can be drawn. Additionally, the inclusion of athletes from multiple sports introduces heterogeneity, which may have influenced variability in neuromuscular performance and postural control. Multiple statistical tests were conducted, increasing the risk of Type I error. Furthermore, data collection across two MCs with repeated measurements would have been desirable to capture MC-related fluctuations more reliably and to better account for both intra- and inter-individual differences. Evaluating only one MC may introduce additional random variation and limit the reproducibility of phase-related findings due to within-person variability. The absence of clear associations between UI or MC phase and risk factors may also partly reflect the strict inclusion criteria. Regarding the CCA, although statistically significant, some coefficients and structure coefficients were counterintuitive. For example, higher well-being scores (indicating worse well-being) were occasionally associated with better performance on risk factors. This is likely attributable to methodological constraints, including low variance in MC-related symptoms, which reduces the stability of canonical coefficients, and the relatively small sample size in relation to the number of variables, which can affect the reliability of the canonical functions.

## 5. Conclusions

This study indicates that the MC phase alone exerts limited influence on ACL injury risk factors in elite female athletes, whereas hormonal fluctuations and, in particular, symptom burden tend to show stronger associations with postural control and hip strength. These findings emphasize the importance of individualized assessment over generalized MC phase-based interpretations. Integrating MC and symptom monitoring into routine athlete management may offer a practical approach to optimize performance, support health, and refine injury-prevention strategies, although these suggestions remain conceptual and require validation in larger studies. Further research involving larger, more diverse cohorts is needed to investigate the complex interactions among hormonal profiles, symptoms, pelvic floor function, and risk factors.

## Figures and Tables

**Table 1:** Descriptive characteristics of the final participant group (*n* = 10).

	Non-UI (*n* = 6)	UI (*n* = 4)
Age, years	25.8 ± 3.3	21.8 ± 2.9
Height, cm	171.0 ± 6.8	164.0 ± 8.6
Weight, kg	68.1 ± 6.4	62.5 ± 8.7
BMI, kg/m^2^	23.3 ± 1.6	23.1 ± 0.9
Experience in main sport, years	18.2 ± 3.3	13.5 ± 5.1
Weekly training volume, hours	11.3 ± 2.3	10.3 ± 0.5
Age at menarche, years	13.8 ± 1.5	11.9 ± 0.3
Menstrual cycle length, days	27.7 ± 1.8	28.3 ± 2.6
Luteal phase length, days	13.0 ± 1.5	13.8 ± 1.7

Abbreviations: BMI, body mass index; UI, urinary incontinence. Data are presented in the form: mean ± SD.

**Table 2:** Saliva sex hormone concentrations, symptoms, training load, sleep, and well-being across the three menstrual cycle phases (*n* = 10).

	EFP	OP	MLP	Effect of MC Phase ^†^ *p*; Effect Size
**Hormone concentrations**				
Estradiol [pg/mL]	4.0 (3.6–4.9)	4.7 (3.3–12.5)	5.3 (3.9–10.9)	0.314; ω = 0.126
Progesterone [pg/mL]	24.4 (15.5–36.6)	61.8 (40.1–91.5)	212.9 (155.3–302.8)	<0.001 *; *ω* = 0.910
Testosterone free [pg/mL]	50.1 (45.8–59.6)	60.7 (56.0–66.0)	56.1 (46.1–63.3)	0.316; *ω* = 0.130
Progesterone:Estradiol Ratio	5.3 (3.4–6.1)	9.9 (7.2–12.6)	35.2 (25.4–43.5)	<0.001 ^‡,^*; η_p_^2^ = 0.782
**Symptoms via PMS questionnaire**				
Overall Score [Min = 0; Max = 81]	8.0 (2.3–20.5)	12.0 (6.3–16.0)	4.0 (0.5–16.8)	0.886; *ω* = 0.020
Cluster Depressiveness	0.2 (0.0–0.6)	0.0 (0.0–0.6)	0.0 (0.0–0.0)	0.687; *ω* = 0.045
Cluster Anxiety	0.2 (0.0–0.7)	0.3 (0.0–0.9)	0.0 (0.0–1.6)	0.880; *ω* = 0.019
Cluster Emotional lability	0.0 (0.0–0.7)	0.2 (0.0–0.7)	0.0 (0.0–0.3)	0.930; *ω* = 0.015
Cluster Irritability	0.0 (0.0–0.0)	0.0 (0.0–0.0)	0.0 (0.0–0.4)	0.556; *ω* = 0.100
Cluster Diminished interest	0.0 (0.0–0.8)	0.0 (0.0–1.8)	0.0 (0.0–0.8)	0.872; *ω* = 0.050
Cluster Concentration problems	0.0 (0.0–0.8)	0.0 (0.0–1.0)	0.0 (0.0–0.0)	0.920; *ω* = 0.018
Cluster Lethargy	0.5 (0.0–1.0)	0.8 (0.0–1.5)	0.8 (0.0–1.0)	0.665; *ω* = 0.046
Cluster Appetite and eating behavior	0.0 (0.0–0.8)	0.0 (0.0–0.9)	0.0 (0.0–0.5)	0.790; *ω* = 0.032
Cluster Sleep behavior	0.0 (0.0–0.8)	0.5 (0.0–1.8)	0.0 (0.0–0.8)	0.798; *ω* = 0.037
Cluster Overwhelm	0.0 (0.0–0.0)	0.0 (0.0–0.8)	0.0 (0.0–0.0)	0.905; *ω* = 0.010
Cluster Physical symptoms	0.3 (0.2–0.5)	0.3 (0.2–0.3)	0.2 (0.0–0.4)	0.543; *ω* = 0.068
**Training load**				
sRPE [AU]	30.0 (0.0–540.0)	210.0 (0.0–967.5)	87.5 (0.0–720.0)	0.332; *ω* = 0.124
Training intensity [Min = 0; Max = 10]	1.5 (0.0–6.0)	3.0 (0.0–7.8)	2.0 (0.0–5.8)	0.342; *ω* = 0.115
Training duration [min]	15.0 (0.0–90.0)	58.0 (0.0–135.0)	17.5 (0.0–101.3)	0.488; *ω* = 0.081
**Sleep**				
Sleep duration, [h:min]	7:30 (7:29–8:30)	7:00 (6:00–7:00)	7:20 (7:00–7:56)	0.062 ^§^; η_p_^2^ = 0.256
**Well-being [1 = very good; 5 = very bad]**				
Overall Score [Min = 5; Max = 25]	11.5 (8.0–13.0)	14.0 (11.0–14.0)	11.5 (9.3–15.8)	0.828; *ω* = 0.019
Sleep quality	2.0 (1.3–3.0)	3.0 (2.3–3.0)	2.0 (1.0–2.8)	0.343; *ω* = 0.116
Muscular condition	2.0 (2.0–2.8)	3.0 (2.0–3.0)	3.0 (2.3–3.0)	0.600; *ω* = 0.057
Physical condition	2.0 (2.0–2.8)	2.5 (2.0–3.8)	3.0 (2.0–3.8)	0.355; *ω* = 0.117
Emotional balance	2.0 (1.3–3.0)	2.0 (2.0–2.8)	2.0 (2.0–2.8)	0.928; *ω* = 0.011
Mental condition	2.5 (2.0–3.0)	2.5 (2.0–3.0)	2.0 (2.0–3.0)	0.800; *ω* = 0.030

Abbreviations: EFP, early follicular phase; OP, ovulation phase; MLP, mid-luteal phase; PMS, premenstrual syndrome; sRPE, session rating of perceived exertion. Data are presented in the form: median (IQR). **^†^** Effect of menstrual cycle phase assessed by Friedman test; ^‡^ repeated-measures ANOVA with Greenhouse–Geisser correction; ^§^ repeated-measures ANOVA; * indicates statistical significance.

**Table 3:** Static and dynamic postural control and hip strength across menstrual cycle phases and UI groups.

	EFP		OP		MLP		Interaction of MC Phase x UI	Effect of MC Phase	Effect of UI	Mean Within-Participant CV
Variable	Non-UI (*n* = 6)	UI (*n* = 4)	Non-UI (*n* = 6)	UI (*n* = 4)	Non-UI (*n* = 6)	UI (*n* = 4)	*p*; Effect Size	*p*; Effect Size	*p*; Effect Size	[%]
Body mass [kg]	67.7 (63.8–70.4)	58.9 (55.8–65.0)	67.5 (64.0–70.0)	60.3 (57.1–64.8)	67.8 (63.7–70.2)	59.8 (57.4–64.8)	0.627; η_p_^2^ = 0.057	0.648; η_p_^2^ = 0.053	0.229; η_p_^2^ = 0.175	1.1
**Static Postural Control**										
**Bipedal stance EO**										
Total sway path [cm]	19.1 (16.1–22.8)	19.0 (17.2–20.5)	17.8 (17.2–18.8)	18.4 (15.3–21.2)	17.9 (15.3–19.9)	18.7 (16.2–21.0)	0.828; η_p_^2^ = 0.084	0.728; η_p_^2^ = 0.118	0.964; η_p_^2^ = 0.000	12.1
Sway area [cm^2^]	0.7 (0.6–0.9)	0.5 (0.4–0.7)	0.7 (0.4–1.2)	0.6 (0.5–0.8)	0.9 (0.6–1.0)	0.7 (0.6–0.8)	0.895; η_p_^2^ = 0.028	0.628; η_p_^2^ = 0.116	0.354; η_p_^2^ = 0.108	37.4
**Bipedal stance EC**										
Total sway path [cm]	26.8 (20.8–34.4)	22.3 (21.3–25.9)	23.8 (21.2–29.6)	26.2 (21.3–30.4)	25.5 (21.6–30.5)	27.2 (23.0–29.0)	0.709; η_p_^2^ = 0.042	0.895; η_p_^2^ = 0.014	0.825; η_p_^2^ = 0.007	10.7
Sway area [cm^2^]	1.1 (0.8–1.5)	0.6 (0.5–0.9)	1.0 (0.8–1.1)	0.8 (0.7–0.9)	1.0 (0.7–1.3)	0.8 (0.7–1.0)	0.538; η_p_^2^ = 0.075	0.819; η_p_^2^ = 0.025	0.327; η_p_^2^ = 0.120	33.6
**Single-leg stance EO**										
Total sway path [cm]	79.4 (67.3–92.4)	75.3 (71.8–81.0)	75.5 (68.9–85.7)	72.2 (66.4–76.5)	76.3 (70.7–83.9)	74.8 (69.7–80.1)	0.709; η_p_^2^ = 0.023	0.894; η_p_^2^ = 0.039	0.825; η_p_^2^ = 0.000	5.8
Sway area [cm^2^]	5.5 (4.8–6.1)	4.2 (3.4–5.5)	5.5 (5.2–5.7)	4.3 (3.4–5.0)	6.1 (5.5–6.8)	4.8 (4.4–5.3)	0.537; η_p_^2^ = 0.013	0.818; η_p_^2^ = 0.055	0.327; η_p_^2^ = 0.108	15.9
**Single-leg stance EC**										
Total sway path [cm]	181.9 (158.0–271.0)	177.1 (141.9–211.0)	156.6 (147.6–187.1)	162.9 (146.9–170.1)	208.6 (178.7–289.3)	161.1 (142.1–170.9)	0.036 *; η_p_^2^ = 0.341			12.3
Sway area [cm^2^]	22.7 (16.3–28.1)	20.0 (13.2–33.6)	20.2 (15.9–26.3)	14.8 (13.3–15.4)	38.7 (18.2–46.6)	13.1 (10.8–14.9)	0.072; η_p_^2^ = 0.280	0.329; η_p_^2^ = 0.130	0.187; η_p_^2^ = 0.207	37.4
**Dynamic Postural Control**										
Anterior difference [cm]	2.0 (1.3–2.8)	5.5 (4.0–8.3)	1.5 (1.0–2.0)	5.0 (2.5–7.8)	1.0 (1.0–1.0)	3.0 (1.5–6.3)	0.533; η_p_^2^ = 0.076	0.113; η_p_^2^ = 0.239	0.077; η_p_^2^ = 0.340	57.4
Composite reach score [%]	90.3 (88.5–91.3)	89.1 (87.5–90.8)	91.3 (88.6–93.5)	90.9 (89.2–91.1)	89.9 (89.2–91.0)	91.3 (90.1–92.3)	0.193; η_p_^2^ = 0.186	0.168; η_p_^2^ = 0.200	0.744; η_p_^2^ = 0.014	1.2
**Hip Strength**										
Abduction strength [N]	351.5 (311.8–375.9)	277.0 (236.4–311.8)	335.5 (299.8–364.1)	277.0 (238.3–315.8)	345.3 (304.1–364.3)	273.5 (242.9–302.3)	0.067; η_p_^2^ = 0.287	0.378; η_p_^2^ = 0.115	0.090; η_p_^2^ = 0.318	2.6
Abduction strength [N/kg]	5.0 (4.4–5.5)	4.3 (4.1–4.5)	4.8 (4.4–5.3)	4.3 (4.0–4.7)	5.0 (4.3–5.4)	4.3 (3.9–4.7)	0.158; η_p_^2^ = 0.206	0.368; η_p_^2^ = 0.118	0.284; η_p_^2^ = 0.142	2.9
Adduction strength [N]	355.0 (339.8–406.3)	328.0 (323.3–390.5)	381.5 (344.3–424.8)	343.0 (330.0–403.0)	368.8 (357.5–407.4)	348.5 (343.0–399.3)	0.959; η_p_^2^ = 0.006	0.481; η_p_^2^ = 0.099	0.939; η_p_^2^ = 0.001	4.0
Adduction strength [N/kg]	5.3 (5.2–5.8)	5.9 (5.8–6.6)	5.3 (5.0–6.1)	6.2 (5.8–6.8)	5.6 (4.9–5.9)	6.3 (6.1–6.8)	0.924; η_p_^2^ = 0.011	0.565; η_p_^2^ = 0.078	0.230; η_p_^2^ = 0.198	4.0
Adduction:Abduction Ratio	1.1 (0.9–1.3)	1.4 (1.4–1.5)	1.1 (1.1–1.2)	1.4 (1.4–1.4)	1.1 (1.0–1.3)	1.4 (1.4–1.5)	0.357; η_p_^2^ = 0.137	0.288; η_p_^2^ = 0.163	0.037 *; η_p_^2^ = 0.487	4.5

Abbreviations: EFP, early follicular phase; OP, ovulation phase; MLP, mid-luteal phase; CV, coefficient of variation; UI, urinary incontinence; EO, eyes open; EC, eyes closed. Data are presented in the form: median (IQR). * indicates statistical significance.

## Data Availability

The dataset from the current study is available from the corresponding author upon reasonable request.

## Supplementary Materials

The following supporting information can be downloaded at: https://www.mdpi.com/article/10.3390/sports14030096/s1, File S1 Daily menstrual cycle monitoring questionnaire in English and German. Figure S1 Individual patterns of single participants during risk factor assessments.

## Abbreviations

The following abbreviations are used in this manuscript:

A	anterior
ACL	anterior cruciate ligament
CCA	canonical correlation analysis
CRS	composite reach score
EC	eyes closed
EFP	early follicular phase
EO	eyes open
ICIQ-UI SF	International Consultation on Incontinence Questionnaire–Urinary Incontinence Short Form
MC	menstrual cycle
MLP	mid-luteal phase
OP	ovulation phase
PL	posterolateral
PM	posteromedial
PMS	premenstrual syndrome
sRPE	session rating of perceived exertion
UI	urinary incontinence
