# Supplementary material for: The Influence of Menstrual Cycle Phase and Urinary Incontinence on Potential ACL Injury Risk Factors with a Focus on Hip Strength and Postural Control in Elite Female Team Sport Athletes: A Pilot Study
Source: Sports (Basel). 2026 Mar 3;14(3):96. doi: 10.3390/sports14030096 (PMC13030537; doi:10.3390/sports14030096)

Figure S1. Individual patterns of single participants during risk factor assessments. (**A**) Static postural con-trol—total sway path; (**B**) Static postural control—sway area; (**C**) Dynamic postural control; (**D**) Hip strength. Light grey represents the non-UI group (n = 6) and dark grey the UI group (n = 4). Dotted lines indicate individual participants, and solid lines indicate the group mean. EC, eyes closed; EFP, early follicular phase; EO, eyes open; OP, ovulation phase; MLP, mid luteal phase.

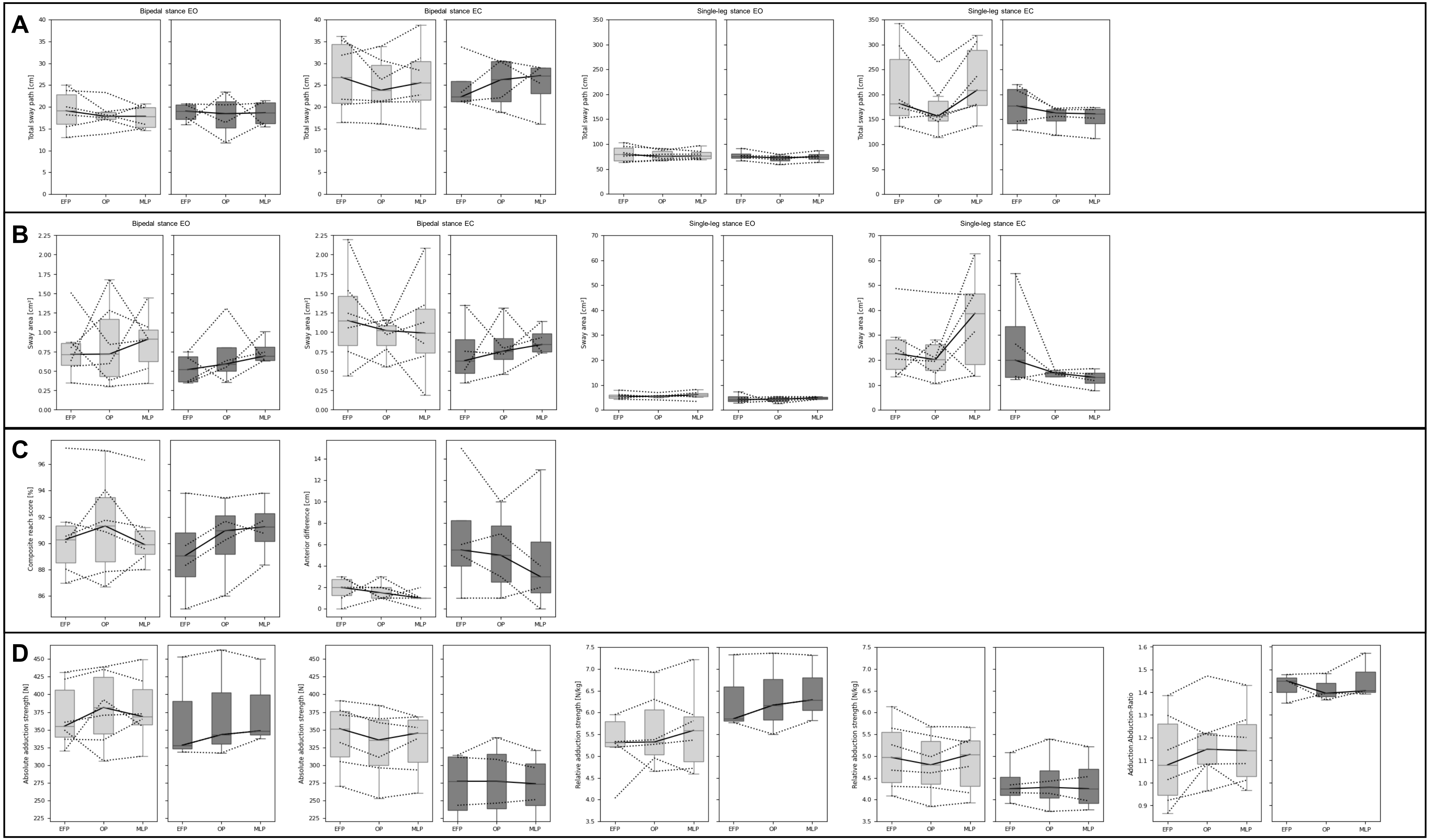

Supplement: Supplementary file 1 [file sports-14-00096-s001.zip › sports-4140246-supplementary Figure S1.pdf]
