# Supplementary material for: The Influence of Menstrual Cycle Phase and Urinary Incontinence on Potential ACL Injury Risk Factors with a Focus on Hip Strength and Postural Control in Elite Female Team Sport Athletes: A Pilot Study
Source: Sports (Basel). 2026 Mar 3;14(3):96. doi: 10.3390/sports14030096 (PMC13030537; doi:10.3390/sports14030096)
Supplement: Supplementary file 1 [file sports-14-00096-s001.zip › sports-4140246-supplementary File S1.pdf]

ESM

Menstrual Cycle Monitoring Questionnaire – German Version

Page 1
1. Hast du heute deine Regelblutung?

- Nein
- Ja, starke Blutung
- Ja, mittlere Blutung
- Ja, schwache Blutung

2. Hast du heute Medikamente eingenommen?

- Nein
- Ja, Schmerzmittel (z.B. Ibuprofen, Paracetamol, …)
- Ja, Erkältungsmedikamente (z.B. Aspririn, ACC akut, WickMediNait, …)
- Ja, sonstige

3. Symptome
im Zusammenhang mit deinem Menstruationszyklus
Mehrfachauswahl möglich

- Bauchkrämpfe/-schmerzen
- Stimmungsschwankungen
- Übelkeit
- Blähungen
- Durchfall
- Kopfschmerzen/Migräne
- Wassereinlagerungen
- Rückenschmerzen
- Andere

Page 2
1. Trainingsbelastung
Wie lange hast du gestern Sport gemacht? [in Minuten]
Freitext

2. Trainingsbelastung
Wie anstrengend war deine Belastung gestern?

- 1 – überhaupt nicht anstrengend
- 2
- 3
- 4
- 5
- 6
- 7
- 8
- 9
- 10 – maximal anstrengend

3. Schlafdauer
Wie lange hast du geschlafen? [in Stunden & Minuten]
Freitext

4. Schlafqualität
Wie hast du geschlafen?

- 1 – sehr gut
- 2
- 3
- 4
- 5 – sehr schlecht

5. Muskulärer Zustand
Wie fühlen sich deine Muskeln an?

- 1 – sehr gut
- 2
- 3
- 4
- 5 – sehr schlecht

6. Körperliche Leistungsfähigkeit
Wie sind deine Energielevel?

- 1 – sehr gut
- 2
- 3
- 4
- 5 – sehr schlecht

7. Emotionale Ausgeglichenheit
Wie ist deine Laune/Stimmung?

- 1 – sehr gut
- 2
- 3
- 4
- 5 – sehr schlecht

8. Mentale Leistungsfähigkeit
Wie ist dein Stresslevel?

- 1 – sehr gut/kein Stress
- 2
- 3
- 4
- 5 – sehr schlecht/sehr gestresst

Page 3
Deutschsprachiger DSM-IV-TR basierter Fragebogen zum prämenstruellen Syndrom

Ditzen, B., Nussbeck, F., Drobnjak, S., Spörri, C., Wüest, D., & Ehlert, U. (2011). Validierung eines deutschsprachigen DSM-IV-TR basierten Fragebogens zum prämenstruellen Syndrom. *Zeitschrift Für Klinische Psychologie Und Psychotherapie, 40*(3), 149–159. https://doi.org/10.1026/1616-3443/a000095

Menstrual Cycle Monitoring Questionnaire – English Version

Page 1

1. Do you have your period today?
   - No
   - Yes, strong bleeding
   - Yes, medium bleeding
   - Yes, weak bleeding

2. Do you took any medication today?
   - No
   - Yes, painkillers (e.g. Ibuprofen, Paracetamol, …)
   - Yes, cold medication (e.g. Aspririn, ACC akut, WickMediNait, …)
   - Yes, other

3. Symptoms

in association with your menstrual cycle
multiple choice possible

- Stomach camps/pain
- Mood changes
- Nausea
- Bloating
- Diarrhea
- Headaches/migraines
- Water retention
- Back pain
- other

Page 2

1. Training load

How long did you exercise (training, games,...) yesterday? [in minutes]
Open-ended

2. Training load

How exhausting was your exercise yesterday?

- 1 – not exhausting at all
- 2
- 3
- 4
- 5
- 6
- 7
- 8
- 9
- 10 – maximal exhausting

3. Sleep duration

How long did you sleep? [in hours & minutes]
Open-ended

4. Sleep quality
How was your sleep?

- 1 – very good
- 2
- 3
- 4
- 5 – very bad

5. Muscular condition
How are your muscles?

- 1 – very good
- 2
- 3
- 4
- 5 – very bad

6. Physical condition
How are your energy levels?

- 1 – very good
- 2
- 3
- 4
- 5 – very bad

7. Emotional balance
How is your mood?

- 1 – very good
- 2
- 3
- 4
- 5 – very bad

8. Mental condition
How are your stress levels?

- 1 – very good/no stress
- 2
- 3
- 4
- 5 – very bad/very stressed

Page 3

German DSM-IV-TR based premenstrual syndrom questionnaire

Ditzen, B., Nussbeck, F., Drobnjak, S., Spörri, C., Wüest, D., & Ehlert, U. (2011). Validierung eines deutschsprachigen DSM-IV-TR basierten Fragebogens zum prämenstruellen Syndrom. *Zeitschrift Für Klinische Psychologie Und Psychotherapie, 40*(3), 149–159. https://doi.org/10.1026/1616-3443/a000095

Translation support in participants' native languages for the German DSM-IV-TR–based premenstrual syndrome questionnaire		
**Danish**	**Spanish**	**Polish**
Mange kvinder rapporterer psykologiske og fysiske ændringer i løbet af deres cyklus. Vi har nedenfor listet eksempler på sådanne ændringer. Besvar venligst, om du normalt bemærker disse ændringer, før din menstruation begynder, og hvor generende disse ændringer er for dig.	*Muchas mujeres muestran a lo largo de su ciclo menstrual cambios psíquicos y físicos. En la siguiente lista se encuentran enumerados algunos de estos cambios. Por favor, conteste si percibe habitualmente alguno de estos cambios antes del comienzo de su período y cómo le afectan los mismos.*	Wiele kobiet zgłasza zmiany psychiczne i fizyczne w trakcie cyklu miesiączkowego. Poniżej wymieniliśmy przykłady takich zmian. Proszę odpowiedzieć, czy zazwyczaj odczuwa Pani te zmiany przed rozpoczęciem miesiączki i w jakim stopniu są one dla Pani uciążliwe.
• Gælderikke<br>• Gælder næsten ikke<br>• Gælder moderat<br>• Gælder stærkt	• No ocurre<br>• Apenas ocurre<br>• Ocurre moderadamente<br>• Ocurre con frecuencia	• Nie zgadza sie<br>• Prawie sie nie zgadza<br>• Zgadza sie czesciowo<br>• Zdecydowanie sie zgadza
1. Jeg er i et mærkbart dårligere humør end normalt	1. Estoy de mucho peor humor que de costumbre	1. Jestem w znacznie gorszym nastroju niż zwykle
2. Jeg føler mig mere håbløs end normalt	2. Me siento más desesperada de lo normal	2. Czuję się bardziej beznadziejnie niż zwykle
3. Jeg føler mig mere værdiløs end normalt	3. Siento que no valgo nada	3. Czuję się bardziej bezwartościowy niż zwykle
4. Jeg oplever mere angst end normalt	4. Tengo más miedo de lo normal	4. Odczuwam większy niepokój niż zwykle
5. Jeg føler mig mere anspændt end normalt	5. Me siento más nerviosa de lo habitual	5. Czuję większe napięcie niż zwykle
6. Jeg har en fornemmelse af at være mere irritabel end normalt	6. Tengo la sensación de estar más alterada que de costumbre	6. Mam wrażenie, że jestem bardziej rozdrażniony niż zwykle
7. Jeg føler mig pludselig trist	7. De repente me siento triste	7. Odczuwam nagly smutek
8. Jeg begynder pludselig at græde	8. Me pongo a llorar sin más	8. Nagle zaczynam płakać
9. Jeg er mere følsom over for afvisning end normalt	9. Soy mucho más sensible al rechazo de lo habitual	9. Jestem bardziej wrażliwy na odrzucenie niż zwykle
10. Jeg er oftere vred end normalt	10. Estoy mucho más enfadada de lo normal	10. Jestem zła częściej niż zwykle
11. Der opstår flere skænderier end normalt	11. Discuto más de lo habitual	11. Częściej niż zwykle dochodzi do klotni
12. Jeg har mindre interesse for ting, jeg ellers nyder (f.eks. arbejde, skole, hobbyer)	12. Me intereso menos por las cosas que me gusta hacer normalmente (p. ej., trabajo, colegio, pasatiempos)	12. Odczuwam mniejsze zainteresowanie rzeczami, które zwykle lubię robić (np. praca, szkoła, hobby)
13. Jeg har en fornemmelse af, at jeg har sværere ved at koncentrere mig end normalt	13. Tengo la impresión de que me cuesta más concentrarme	13. Mam wrażenie, że mogę się gorzej niz zwykle skoncentrować

14. Jeg bliver hurtigere træt end normalt	14. Me canso antes de lo habitual	14. Męczę się szybciej niż zwykle
15. Jeg har mindre energi end normalt	15. Tengo menos energía de lo normal	15. Mam mniej energii niż zwykle
16. Mit spisemønster er anderledes (jeg spiser mere eller mindre) end normalt	16. Mi conducta alimentaria es diferente (como más o menos de lo normal)	16. Moje zachowania żywieniowe są inne (jem więcej lub mniej) niż zwykle.
17. Jeg har lyst til anden mad end normalt	17. Tengo más ganas de comer otros alimentos que no son los habituales	17. Mam apetyt na inne pokarmy niż zwykle.
18. Mit søvnbehov er anderledes (jeg sover mere eller mindre) end normalt	18. Mi necesidad de sueño es diferente (duermo más o menos de lo normal)	18. Moje zapotrzebowanie na sen jest inne (śpię mniej lub więcej) niż zwykle.
19. Jeg har en følelse af at have mindre kontrol end normalt	19. Tengo la sensación de no tener las cosas bajo control	19. Mam wrażenie, że mam mniejszą kontrolę niż zwykle
20. Jeg føler spænding i brystet	20. Siento tensión en las mamas	20. Czuję ucisk w klatce piersiowej
21. Mine bryster er mere ømme end normalt	21. Mis mamas son más sensibles de lo normal	21. Moje piersi sa bardziej wrażliwe niż zwykle
22. Mine bryster føles hævede	22. Mis senos están inflamados	22. Moje piersi sa nabrzmiale
23. Jeg har hovedpine	23. Tengo dolor de cabeza	23. Boli mnie głowa
24. Jeg har ledsmerter	24. Me duelen las articulaciones	24. Mam bóle stawów
25. Jeg har muskelsmerter	25. Tengo dolor muscular	25. Odczuwam ból mięśni
26. Jeg føler mig tykkere end normalt	26. Me siento más gorda de lo normal	26. Czuję się grubsza niż zwykle
27. Jeg vejer mere end normalt	27. Peso más de lo normal	27. Ważę więcej niż zwykle
Disse symptomer påvirker mig …	Estos síntomas...	Objawy te dotyczą mnie ...
• Slet ikke<br>• Næsten ikke<br>• Moderat<br>• Stærkt	• No me afectan<br>• Apenas me afectan<br>• Me afectan moderadamente<br>• Me afectan mucho	• Wcale<br>• Mało<br>• Umiarkowanie<br>• silne
28. … på arbejdspladsen/i skolen	28. ... en el trabajo/en el colegio	28. ... w miejscu pracy / w szkole
29. … i sociale aktiviteter	29. ... en actividades sociales	29. ... w działalności społecznej
30. … i relationer til andre	30. ... en mis relaciones con los demás	30. ... w relacjach z innymi
